# Evidence of Signs and Symptoms of Craniomandibular Disorders in Fibromyalgia Patients

**DOI:** 10.2174/1874210601711010091

**Published:** 2017-02-14

**Authors:** Massimo Corsalini, Daniela Di Venere, Biagio Rapone, Gianluca Stefanachi, Alessandra Laforgia, Francesco Pettini

**Affiliations:** Dental School - University of Bari, Piazza Giulio Cesare, Bari, Italy

**Keywords:** Craniomandibular disorder, Fibromyalgia patient, Gnathologic examination, Hypothyroidism, Myofascial pain

## Abstract

**Objective:**

The purpose of this study is to highlight the evidence of signs and symptoms of craniomandibular disorders (CMD) in patients suffering from fibromyalgia.

**Materials and Method:**

The study has been carried out from May 2011 to May 2015, recruiting a sample of fibromyalgia patients at the Department of Neurophysiopathology at the hospital Policlinico in Bari. Among the 150 examined patients, 60 of them have been diagnosed to suffer from fibromyalgia and 27 accepted to be investigated with a gnathologic examination at the Dental School at the University of Bari.

**Results:**

24 patients (88.9%) were women and 3 (11.1%) men; from 26 to 66 years old (average age, 39). 14 patients (51.9%) were affected by primary fibromyalgia, the remaining 13 (48.1%) by secondary fibromyalgia, mainly associated with hypothyroidism (29.6%). VAS average score was about 8 ± 1.85. The frequency of pain was daily in 15 patients (55.6%); twice a week in 10 patients (37.03%) and a few times a month in 2 patients (7.4%). 11 patients (40.7%) attributed the onset of fibromyalgia to a specific instigating event. In addition, from the gnathologic anamnesis, 11 patients (40,7%) reported a painful symptom in the head-neck region, especially in the frontal region, in the neck, in the masseter muscle and ATM. VAS average score was 3.4 ± 2.8, significantly lower than the one referring to the fibromyalgia pain. The gnathological examination found CMD signs and symptoms in 18 patients (66.7%). Concerning the prevalence of CMD, in type I fibromyalgia, myofascial pain was more frequent (5 patients), whereas in type II fibromyalgia, what was more frequent was a dislocation with reduction (3 patients).

**Conclusion:**

Based on clinic experience, we can affirm that some patients with CMD report pain in other regions. It is difficult to distinguish the CMD forms directly correlated to fibromyalgia from those engendered by parafunctional activities; hence the need is to resolve the fibromyalgia syndrome adopting a multidisciplinary approach.

## INTRODUCTION

Fibromyalgia [1-3] is a form of extra-articular rheumatism or soft tissues rheumatism, characterised according to the American College of Rheumathology (ACR) [2], by at least 3 months of diffuse pain and tenderness in at least 11 of 18 muscle-skeletal points localised in nine regions of the body. We distinguish two types of fibromyalgia: primary fibromyalgia, where laboratory exams are not altered and there is not any association with a well-known disease; secondary fibromyalgia, where symptoms emerge in the course of other pathologies also defined as “main pathologies” (rheumatic pathologies, chronic infection and inflammations, endocrine disorders).

Fibromyalgia has a prevalence of 2%, mainly in the female gender (3.4% *vs.* 0.5%) with an onset peak between 45 and 60 years of age [4].

Etiology of fibromyalgia is multifactorial [5]. Different risk factors have been identified. Among those, some are socio-cultural (high prevalence in low-income population, poor education level), depression, anxiety, muscle-skeletal pain and traumas.

The predominant symptom in all fibromyalgia patients is a diffuse pain [6, 7] which can be reported as a sensation of burning, stiffness, contracture and tension; it is persistent, diffuse, deep, excruciating and it can vary depending on the time of the day, the activity levels, weather conditions, sleep cycle and stress. Diffuse pain is measured with specific questionnaires using VAS scale (visual-analogic pain scale).

Craniomandibular disorders (CMD) are a series of clinical dysfunctions involving masticatory muscles, temporomandibular joint (TMJ) or both; there is a strict correlation with other muscle-articular structures of the head and neck and with the postural stance [8].

A physical diagnosis (axis I) and an evaluation of the social and psycho-affective involvement (axis II) are classified according to the RDC/TDM (Research Diagnostic Criteria for Temporomandibular Disorders) [9]. The physical diagnosis provides three sub-groups: muscle pathologies, discopathies; arthralgia, arthritis, arthrosis; axis II measures the severity of pain, related disability and distress and the psychological condition; a serious psychological involvement may lead to an interdisciplinary treatment because these are cases where the chances of failure are higher.

The signs and symptoms of CMD are widespread in adult population with a prevalence of the formers and an average value of 44% against 30% of the latter [8], but they can also be found in childhood and in teenage.

The main signs and symptoms are muscle pain in the head and neck, noise and pain in the temporomandibular joint, alteration of the mandibular mobility; further symptoms are cephalea, facial and nape pain, dental pain, tinnitus dysgeusia.

CMD has a multifactorial genesis. The pain is determined by a complex interaction between mechanical, biological, cognitive and psycho-emotional factors. In addition, there are perpetuating, instigating and predisposing factors.

The predisposing factors include morphostructural alterations (anatomical alterations), psycho-emotional (stress, anxiety) and pathophysiological alterations (para-functions).

The instigating factors include overload, micro and macro-traumas. Perpetuating factors are to be found in the psycho-emotional dimension of the patient (emotional, behavioural and social factors) [10].

The most frequent symptom is pain [11]; CMD is the most frequent cause of non-odontogenic facial pain.

CMD treatment can be causal or symptomatic, taking into consideration the symptoms, severity and duration of the pathology.

Both fibromyalgia and persistent pain associated to some cases of CMD have long been considered as chronic muscle-skeletal pain conditions; the comorbidity of the two pathologies appears therefore evident.

Even though the craniofacial region is not included by ACR criteria in the diagnosis of fibromyalgia, it is reasonable to think that diffuse pain can affect this region. The first studies concerning this matter showed that fibromyalgia patients could show symptoms associated to some types of CMD, like pain in the masticatory muscles and TMJ; furthermore, some patients with CMD complain a more diffuse pain, extending beyond the craniofacial region.

One of the first studies on the correlation between fibromyalgia and CMD dates back to 1988 [12]: this study shows that fibromyalgia patients can have symptoms and signs of CMD; severe symptoms of mandibular dysfunction were found as well. In all patients, pain was reported at the palpation of the neck and masticatory muscles. We can affirm that fibromyalgia could play an important role in the etiology of CMD; the secondary hyper-reactivity of masticatory muscles causing pain could find an explanation in the physical and psychological tension due to severe symptoms of fibromyalgia.

It has been observed that a wide percentage of subjects with localised myofascial pain in the orofacial region also report pain in other body regions, and that the 20% of patients with signs and symptoms of CMD are positive to ACR criteria for the diagnosis of fibromyalgia. For this reason, some authors inferred that fibromyalgia and myofascial pain in the masticatory muscles are different manifestations of a single syndrome picture. Based on these considerations, a study [13] compared a group of fibromyalgia patients with one affected by CMD and found a strict association between the presence of fibromyalgia and CMD. A high percentage of fibromyalgia patients (86.7%) resulted as being positive for at least one diagnosis of RDC/TMD. In opposition, only a little part of DTM patients (10%) was positive to ACR criteria for the diagnosis of fibromyalgia. This result further confirms that fibromyalgia could be an etiologic factor of CMD.

Considering that pain is a symptom both in fibromyalgia and CMD, the present study aims to describe the involvement of masticatory muscles and temporomandibular joint in fibromyalgia patients according to the RDC/TDM, analysing a group of these patients.

## MATERIALS AND METHODS

The study has been carried out between May 2011 and May 2015, recruiting a sample of fibromyalgia patients at the Department of Neurophysiopathology at the Policlinico hospital in Bari.

Among the 150 examined patients reporting chronic pain not attributed to any pathology, 60 were diagnosed fibromyalgia and 27 accepted to undergo a gnathological examination.

In agreement with the criteria of the American College of Rheumatology (ACR) [2], the inclusion criteria included:

- Diffuse pain for at least 3 months;
- Pain in at least 11 among the 18 tender points after digital palpation in 9 body regions.

For each member of the sample, we recorded age, gender, job and we determined the type of fibromyalgia distinguishing between:

- Primary: there are not any laboratory tests alterations nor association with other diseases;
- Secondary: it emerges during the course of another disease defined “main disease” (rheumatic diseases, chronic infections, endocrine disorders).

We performed a neurological control visit and then we carried out, at Dental School at the University of Bari, a gnathological evaluation in order to verify the presence of diffuse pain in the orofacial region and signs of CMD. The gnathological evaluation was performed in agreement with the protocol defined by the European Academy of Craniomandibular Disorders. This protocol includes the compilation of a dedicated medical record, made up of an anamnestic questionnaire and an objective examination. The anamnesis consists of the patient’s personal data compilation, the assessment of their overall health status, followed by a specific investigation of the reported symptoms of CMD, such as pain in face, head and neck, presence of articular noise (click/crackling), disorders of the mandibular function (opening/closing, during chewing, mandibular stiffness). The objective examination was carried out making the patient undergo a series of examinations and orthopaedic tests with the aim to examine the mandibular dynamics (possible limitations and/or deviations/deflections) and to detect possible pain (during mandibular movements or after the palpation of masticatory muscles and/or TMJ), dental abrasion facets due to parafunctions and articular noises.

At the end of this diagnostic protocol, the patients were classified according to the RCD/TMD criteria [9] in one of the following categories:

- **Group 1:** Muscle pathologies

- 1a: myofascial pain
- 1b: myofascial pain with partial opening

- **Group 2:** Disc dislocations

- 2a: disc dislocation with reduction
- 2b: disc dislocation without reduction, with partial opening
- 2c: disc dislocation without reduction, without partial opening

**- Group 3:** Arthralgia – arthritis – arthrosis

- 3a: arthralgia
- 3b: osteoarthritis of ATM
- 3c: osteoarthrosis of ATM

In the follow-up checks, the neurologist asked the patient about possible change in symptoms and improvement due to the treatment. Pain was measured with VAS scale score points, and then matched with the value reported in the previous checks; after this, the palpation of tender points examination was repeated.

The intensity was examined with the Visual Analogue Scale (VAS), with an arbitrary score ranging from 0 to 10, where 0 is absence of pain and 10 is the maximum pain experienced by the patient. Patients were asked to describe the maximum pain they had experienced and to indicate where they would locate the intensity of the present pain in the scale, giving it a proportional score.

## RESULTS

In Table **1**, we have reported the demographic and clinical data of fibromyalgia in the examined patients.

We recruited 27 patients. 24 patients (88.9%) were women and 3 (11.1%) men; from 26 to 66 years of age (average age, 39). 14 patients (51.9%) were affected by primary fibromyalgia, the remaining 13 (48.1%) by secondary fibromyalgia, mainly associated with hypothyroidism (29.6%). VAS average score was about 8 ± 1.85; the frequency of pain was daily in 15 patients (55.6%), (37.03%) twice a week in 10 patients and (7.4%) a few times a month in 2 patients.

Most patients were assessed according to their socio-economic sphere and as it is evident from the table most of them did not have a job either because they were housewives (48.1%) or because they had left their job because of the limitations due to their pathology.

Starting from the anamnesis, we inferred the characteristics of pain reported by the patient: in those cases where the VAS score was low, the pain was described as a tingling sensation; higher scores corresponded with burning, piercing pain with a sensation of needle sting, stabbing or electric shock, often together with fatigue and weakening.

Almost all patients reported that their pain worsened depending on changes in weather conditions or after mild physical activity (having a walk), but above all in stressful conditions.

In Table **2**, we report the data relative to the specific gnathological anamnesis.

Based on these data, it emerges that 11 patients (40,7%) reported a painful symptomatology in the head-neck region.

The VAS was on average 3.4 ± 2.8, significantly lower than the one reported for fibromyalgia pain. 10 patients (37.03%) reported the CMD symptoms after the diagnosis of fibromyalgia, while 1 patient (3.7%) reported before.

13 patients (48%) reported the presence of parafunctional habits. 8 patients (29.62%) were under treatment with occlusal plate.

In the 18 patients (67%) where it was possible to detect symptoms and signs of CMD, the following sub-groups were identified:

- 6 with disc dislocation with reduction (classified as 2a);
- 1 with disc dislocation without reduction (2b);
- 7 with myofascial pain (1a);
- 3 with myofascial pain and disc dislocation with reduction (1a+2a);
- 1 with myofascial pain and disc dislocation without reduction (1a+2b).

Concerning the prevalence of CMD in two fibromyalgia subgroups, in type I fibromyalgia, we found a more frequent presence of myofascial pain (5 patients), while in type II fibromyalgia, the disc dislocation resulted as more frequent (3 patients).

## DISCUSSION

Despite the fact that the head-neck region is not included in the ACR criteria for the diagnosis of fibromyalgia, it is reasonable to assume that it is affected by diffuse pain involving the rest of the body.

A study on 191 fibromyalgia patients found that 94% of them complained of localised pain in the temporomandibular region [14].

A study examined the prevalence of signs and symptoms of CMD in fibromyalgia and reported that in 53% of the fibromyalgia examined patients, the presence of CMD was seen [15].

Similarly, considering our experience, we can affirm that some CMD patients complain pain in other regions [16, 17].

In the present study, we found that 67% of fibromyalgia patients showed signs and/or symptoms of CMD, mainly referable to the sub-groups of myofascial pain (38,9%) and disc dislocation with reduction (22.3%); none of the patients resulted to belong to the third group.

The explanation of the higher evidence for these two types of CMD could lie in the etiopathogenetic mechanism of fibromyalgia, which leads to the chronicization of a disorder, in most cases self-limiting in time.

In fibromyalgia patients, the transmission path of pain is altered with consequent facilitation and/or failure of the inhibition of the nociception resulting in an increase in sensitivity and in the perception of pain at a central level. This affects the dynamic balance of the whole system, which passes from a state of physiological adaptation to a dysfunctional and clearly pathological one. These modifications are reflected also in the stomatognathic system causing localised pain burdening the masticatory muscles and limiting mandibular movements. The pain can be further exacerbated by concurrence with stress and depression, which often is associated with fibromyalgia although the patient, in most cases, tends to underrate craniomandibular problems perceived as of secondary importance and not associated with the main pathology. VAS average score for CMD is 3.4 ±2.8, below the 8±1.85 of patients with fibromyalgia syndrome.

A study [18] found that a high percentage (75%) of fibromyalgia patients met RDC criteria for most types of CMD, a way lower percentage (18%) of patients with CMD met ACR criteria for fibromyalgia; only a low percentage (20%) of patients with fibromyalgia who met the criteria for CMD asked for a treatment of their state.

A study on patients with fibromyalgia [19] showed that the most common signs are pain in masticatory muscles, in the TMJ, articular click in jaw opening; the aforementioned study defined cephalea and facial pain as symptoms; moreover, the prevalence of; myofascial pain was found.

Given the different time of onset of diffuse and localised pain in the orofacial region, it has been suggested that fibromyalgia may affect first other body segments and then reach the head-neck region.

Most of our patients report the onset of head-neck region upsets later than other body parts; this leads us to assume that fibromyalgia emerges first in other regions and then becomes an instigating factor of CMD.

It is therefore important to understand if facial pain emerges before or after the fibromyalgia syndrome, because it could be an associated comorbidity factor or an instigating factor of fibromyalgia, also, in the light of the fact that in patients with CMD, we can report pain outside the temporomandibular region. Many of the patients with fibromyalgia can develop inflammatory-degenerative disorders affecting the ATM and masticatory muscles, highly likely to be caused by a secondary effect of muscle pain; in the light of these observations, the study suggests that fibromyalgia can be a medium-term, long-term risk factor for CMD.

A study [20] investigated the relation between fibromyalgia and CMD and the role of masticatory dysfunction in both disorders, pointing out that masticatory myofascial pain is an importing factor for the coexistence of fibromyalgia and CMD.

Some authors [21] argued that, although the majority of cases of CMD are mild and self-limiting, around 10% of CMD patients could develop severe disorders associated with pain and chronic disability. It was suggested that fibromyalgia and diffuse pain have a significant role in the chronicization of CMD. This perspective showed an increased risk for the onset of clinically significant pain in CMD when the subjects are affected by fibromyalgia. Based on the results of the study, we can affirm that fibromyalgia and diffuse pain are risk factors complicating the management of CDM and they therefore have to be taken into consideration in both the evaluation and the treatment of CMD. Not recognizing the presence of fibromyalgia or other comorbidity conditions can inevitably lead to a treatment failure, hence it is highly needed for patients to be assisted by a team of specialists to face the different aspects of this pathology.

## CONCLUSION

Based on the results of the study, we can affirm that fibromyalgia and diffuse pain are risk factors complicating the management of CDM and they therefore have to be taken into consideration in both the evaluation and the treatment of CMD.

In our study, we found that 67% of fibromyalgia patients showed signs and/or symptoms of CMD, mainly referable to myofascial pain (38,9%) and disc dislocation with reduction (22.3%). Fibromyalgia may lead to the chronicization of a disorder, in most cases self-limiting in time. The pain can be further exacerbated by concurrence with stress and depression, which often is associated with fibromyalgia although the patient, in most cases, tends to underrate craniomandibular problems perceived as of secondary importance and not associated with the main pathology.

It is however difficult to distinguish the CMD forms directly correlated to fibromyalgia pathology from those due to parafunctional activities, which were present in 48% of the sample; in opposition, in patients under treatment with occlusal plate, any improvement in symptoms has not been seen. Hence, a resolution of fibromyalgia syndrome adopting a multidisciplinary approach is required.

In order to obtain more reliable results, it is necessary to study a wider sample of patients and then match the obtained data with a control group.

## Figures and Tables

**Table 1:** Demographic and clinical data of patients with fibromyalgia.

**Patient**	**Sex**	**Age**	**Type of fibromyalgia**	**Job**	**VAS**	**Pain frequency**
1	F	40	F2	Housewife	10	Weekly
2	F	26	F2	Student	8	Daily
3	F	52	F2	Housewife	7	Daily
4	M	54	F1	Unemployed	10	Daily
5	F	45	F1	Housewife	5	Weekly
6	F	41	F1	Housewife	10	Daily
7	F	66	F1	Dealer	10	Daily
8	F	54	F1	Office worker	7	Weekly
9	F	51	F1	Office worker	5	Daily
10	F	35	F1	Housewife	5	Monthly
11	F	36	F1	Housewife	10	Weekly
12	F	47	F2	Office worker	6	Daily
13	F	49	F1	Housewife	10	Daily
14	F	30	F2	Housewife	6	Monthly
15	M	55	F1	Lawyer	5	Weekly
16	M	59	F1	Pensioner	7	Daily
17	F	51	F2	Housewife	7	Weekly
18	F	62	F2	Housewife	8	Daily
19	F	55	F2	Housewife	9	Weekly
20	F	45	F2	Housewife	8	Daily
21	F	54	F1	Housewife	7	Weekly
22	F	46	F2	Housewife	5	Weekly
23	F	47	F2	Housewife	7	Weekly
24	F	29	F1	Student	8	Daily
25	F	59	F2	Teacher	10	Daily
26	F	43	F1	Office worker	10	Daily
27	F	43	F2	Housewife	9	Daily

**Table 2:** Data relative to the specific gnathological anamnesis.

**Patient**	**Facial pain**	**VAS**	**Site of pain**	**Bite**	**Parafunction**	**EACD grups**
1	+	8	Neck	+	Bruxism	1a
2	+	5			Bruxism	2a
3	+	6	Masseter (right)		Bruxism	1a
4		0	Masseter (right and center), neck			2b
5		0			Bruxism	2a
6	+	10		+		1a
7	+	8		+		1a
8		0				
9	+	3		+	Bruxism	1a
10		0		+		2a
11	+	7			Bruxism, onicofagia	1a,2b
12		0				
13	+	8	Neck, temporalis (right and center), masseter (right and center)		Bruxism	1a, 2a
14		0				
15		0				
16		0				
17		0	Masseter (center), neck		Bruxism	2a
18		0				
19	+	8	Temporalis (right and center), Masseter (center), neck	+	Bruxism	1a
20		0				
21		0				
22		0				
23		0	Temporalis (center), Masseter (center), neck		Bruxism	2a
24	+	7	Temporalis (right and center), masseter (right and center), neck,		Bruxism	1a, 2a
25		0		+		2a
26	+	10	Masseter (right and center), neck	+	Bruxism	1a
27		0	Temporalis (right and center), masseter (right and center), neck		Bruxism	1a, 2a
